# Responsibility and the recursion problem

**DOI:** 10.1111/rati.12327

**Published:** 2021-11-18

**Authors:** Ben Davies

**Affiliations:** ^1^ Oxford Uehiro Centre for Practical Ethics University of Oxford Littlegate House, St Ebbe’s Street Oxford OX1 1PT UK

**Keywords:** applied ethics, egalitarianism, political philosophy

## Abstract

A considerable literature has emerged around the idea of using ‘personal responsibility’ as an allocation criterion in healthcare distribution, where a person's being suitably responsible for their health needs may justify additional conditions on receiving healthcare, and perhaps even limiting access entirely, sometimes known as ‘responsibilisation’. This discussion focuses most prominently, but not exclusively, on ‘luck egalitarianism’, the view that deviations from equality are justified only by suitably free choices. A superficially separate issue in distributive justice concerns the two–way relationship between health and other social goods: deficits in health typically undermine one's abilities to secure advantage in other areas, which in turn often have further negative effects on health. This paper outlines the degree to which this latter relationship between health and other social goods exacerbates an existing problem for proponents of responsibilisation (the ‘harshness objection’) in ways that standard responses to this objection cannot address. Placing significant conditions on healthcare access because of a person's prior responsibility risks trapping them in, or worsening, negative cycles where poor health and associated lack of opportunity reinforce one another, making further poor yet ultimately responsible choices more likely. It ends by considering three possible solutions to this problem.

## 1 INTRODUCTION

In philosophical writing on healthcare, there is considerable discussion of ‘personal responsibility’ in healthcare allocation (Buyx, 2008; Friesen, 2018; Le Grand, 2014; Levy, 2018; Savulescu, 2018; Sharkey & Gillam, 2010; Wikler, 2002). Sometimes called ‘responsibilisation’ (Brown et al., 2019), this debate sits within the broader context of responsibility–sensitivity in distributive justice, i.e., the extent to which individual entitlements depend on prior free choices. A prominent worry about responsibilisation is the ‘harshness’ objection (Albertsen & Nielsen, 2020; Anderson, 1999; Fleurbaey, 1995; Voigt, 2007), that responsibility–sensitive policies imply excessively punitive consequences for those who are responsible for their (health) needs.

Paradigm discussions of harshness focus on one–off choices, e.g., traffic incidents that leave careless drivers at risk of death if untreated (Anderson, 1999; Fleurbaey, 1995). While some broader work on healthcare responsibility considers the longer–term (Brown & Savulescu, 2019), there is still a focus on agents’ future *health*. This paper engages with an under–explored issue by highlighting what may seem obvious, but which has received little explicit attention: the potential for responsibilisation to trap individuals in negative feedback loops, or to exacerbate the situation of those already trapped in such loops, an issue I call the ‘recursion problem’. A corollary of the problem is that when we talk about holding individuals substantively responsible by ensuring that they bear the costs of their choices, we must acknowledge just how far–reaching those costs may be.

Responsibility–sensitivity is primarily discussed in the literature on luck egalitarianism, a family of views emphasising the importance of ideas including choice, luck, responsibility, control, and desert (Lippert‐Rasmussen, 2016, pp. 55–76). Luck egalitarians assign a ‘distributive significance…to the distinction between the chosen (associated with responsibility and the voluntary) and the unchosen (luck, or the absence of the voluntary)’ (Albertsen & Knight, 2015, p. 165). This is often cashed out in the view that ‘considerations of responsibility can excuse departures from strict equality’ (Vincent, 2009, p. 39), though there may be other reasons to oppose such departures (Cohen, 2008, p. 381; Segall, 2010, p. 69). Still, responsibility–sensitivity is not tied to egalitarianism; one might insist of any distributive principle that deviations from it are justified by appropriate choices. Many who do not embrace the mantle of luck egalitarianism think responsibility has some role in distributive questions (Dworkin, 2013, p. 226; Honoré, 1999, p. 134; Scanlon, 2019). I focus on luck egalitarianism given its dominance; but my discussion applies more widely.

Luck egalitarians are not concerned only with healthcare, but this is where the debate around responsibility tends to occur. Justifications for responsibility–sensitivity depend on various considerations, but a central one appeals to the idea that if I am suitably responsible for a choice, it is unfair if others bear the foreseeable costs of that choice. A quite separate, though consistent, idea appeals to the positive effects of holding people responsible, such as the potential to make individuals more likely to act responsibly in the future (McGeer, 2019).

A superficially separate issue is the relationship between health and other social goods. The literature on the ‘social determinants of health’ (Braveman & Gottlieb, 2014; Marmot & Wilkinson, 2006; Marmot et al., 2020) notes the influence of social goods other than medical care on individual health. Health also influences individual opportunities (Daniels, 2008) and capabilities (Powers & Faden, 2008; Venkatapuram, 2011). Thus, there is a two–way, often mutually reinforcing effect between health and prospects in other areas.

Section 2 expands on this two–way relationship and suggests some initial issues it raises for luck egalitarianism. Section 3 turns to how this fact interacts with the idea of responsibilisation, and to the potential for a deeper problem: that responsibilisation policies may mean a single poor choice has extreme effects. Thus, the stakes of holding people responsible in healthcare are potentially significantly higher than is often acknowledged. This is the ‘recursion problem’. I end in Section 4 considering potential solutions.

An initial caveat: My argument focuses on a fairly unconstrained form of responsibilisation, which insists on individuals bearing *at least* all the unavoidable costs of their choices. A cost is unavoidable when it, or some equivalent, must be borne by someone. The direct effect on health of refusing someone medical care is often avoidable: if we treat Vikram's broken leg, nobody necessarily bears the health cost that would have arisen had we refused to do so (though in cases of triage, someone might). However, the disjunctive cost of those health burdens *or* the financial cost of treating Vikram is unavoidable: somebody must bear one or the other of these costs, at some point.

An obvious retort is that nobody endorses unconstrained responsibilisation. My aim, however, is to consider which constraints, if any, can resolve the recursion problem while respecting the core commitments of responsibilisation. Starting with an account that assumes particular constraints would be unhelpful.

## 2 HEALTH AND OPPORTUNITY

Responsibility is intimately linked to opportunity. That you had no control over, or opportunity to avoid, an outcome is often, though not always (Frankfurt, 1969), an excuse. Moreover, you are generally less responsible for outcomes which are more difficult or more costly to avoid. A choice is *difficult* when it is hard to make, and *costly* when its negative effects are considerable. Gambling all my savings on the lottery is all too easy, but likely costly. Eating a nutritionally balanced diet is difficult for someone who lacks access to relevant information but is not *thereby* made costly for that individual.

Opportunities influence health. This fact has been thoroughly explored in discussions of responsibilisation (Brown et al., 2019; Cappelen & Norheim, 2005; Feiring, 2008); luck egalitarianism has received criticisism based on the social determinants of health, though as Albertsen (2015) outlines, this may conflate the theoretical idea of responsibility–sensitivity with specific policies. As well as effects of social disadvantage that bypass individual choice entirely, (Deaton, 2013; Marmot & Wilkinson, 2006), deprivation may make it more difficult or costly to choose well (Levy, 2019). For instance, those who must work multiple jobs have less time and energy to make positive dietary choices. They are not unable to do so, but it is harder. Those with limited money face greater costs for some health–promoting choices. At an extreme end, some must choose between food and heating, so that either health–promoting choice also has a high health cost. If the effect of opportunity on health were all–encompassing, this might undermine the case for responsibilisation altogether. Yet as Schwan (2021) notes, that a person's health is *partially* determined by their social situation need not mean they are incapable of being responsible for health needs that result from their choices, though it may place limits on how, or whether, others should hold them responsible.

While many discussions of responsibilisation in health focus on healthcare alone, Albertsen and Knight (2015) note that this seems unjustified given the relationships between health and other goods (see also Voigt, 2013, p. 147). They advocate, *inter alia*, compensating people's unchosen and untreatable shortfalls in health by benefitting them in other ways. But these connections generate a broader issue for luck egalitarianism, that a choice which directly affects your health will probably affect other areas of your life. If a person's employment, education, or housing deficits are partly caused by health deficits, and those health deficits come from free choices, luck egalitarians must either regard those other inequalities as ‘chosen’ or explain why the moral power of choice does not transfer to predictable effects.

Health also influences opportunities. This is sometimes called the ‘latent positionality’ of health, referencing the idea that while health is intrinsically valuable, it has a less obvious ‘competitive value’, since ‘healthy people are…more likely to succeed in the competition for jobs and other scarce goods’ (Brighouse & Swift, 2006, p. 479). Someone with complex health needs faces barriers to securing reasonably paid work or completing education. This may affect their ability to access good quality housing and other amenities. Thus, there is a dual, potentially mutually reinforcing relationship between health and other opportunities and social goods.

This relationship is not easily ‘de–positionalised’ by restricting consumption. Ben Shahar (2018, p. 112) suggests that one method of de–positionalising is to ‘remove end–use goods from the market’, citing the way organs for transplantation are allocated by a ‘combination of need, expected utility, and waiting time’. Yet as Simmerling (2007, p. 442) notes, at least in the United States ‘the reality is that the uninsured, underinsured and the poor do not currently have an equal opportunity to fully realize the benefits of organ transplantation because they do not have equal access to…post–transplant immunosuppressant medications’. Even in apparently ‘de–positionalized’ areas, it is difficult to anticipate and prevent how social position impacts health. Moreover, to reiterate, to the extent that inequality of access is a result of prior free choices against an equitable background, it is unclear that luck egalitarians have the conceptual resources to justify such de–positionalizing, at least taken from within the theory.

Advocates of responsibility–sensitivity can answer many pragmatic issues raised by the social determinants of health by noting that to the extent that a health need is socially determined, it is not part of someone's choice and thus not something for which luck egalitarianism should hold them responsible. The two–way relationship between health and opportunity, however, creates a special problem. An initial choice, even one made under fair conditions, may have a considerable influence over an individual's lifetime due to recursive interplay between health's effects on opportunities and opportunities' effects on health. This interplay may occur without responsibilisation, but responsibilisation exacerbates it in two ways. It means more individuals are likely to be drawn into this recursive cycle because more individuals will be made to bear the significant initial costs of health–affecting choices. And responsibilisation means that even those who might have suffered from this negative cycle in a responsibility–insensitive healthcare system are likely to suffer from it to a greater extent.

## 3 THE RECURSION PROBLEM

I have mentioned the harshness objection, which focuses on an apparent willingness to treat people very harshly when their need is generated by suitably situated choice. Luck egalitarians offer various responses (Knight, 2015; Segall, 2010; Stemplowska, 2013), two of which are particularly relevant.

Bærøe and Cappelen (2015) note that responsibility–sensitivity need not entail treatment denial; we could hold people responsible by raising taxes on certain behaviours which then fund care (see also Albertsen & Knight, 2015, p. 168), though this cannot cover health needs which are caused by *failure* to do certain things, such as exercise. It is also worth noting that if, as I suggested above, a primary motivation for responsibilisation is the thought that others should not cover the costs of my free choices, this seems only to justify insisting that I cover the unavoidable costs of those choices. Some costs only arise if other costs are not met. Recall Vikram. His health needs generate two sets of costs: direct health costs, and financial costs. Some of Vikram's health costs will only arise if he goes untreated. Thus, they are avoidable. However, the financial costs involved in treating him are unavoidable. This has implications for responsibilisation: the justification that relies on others not bearing the costs of my choices cannot directly recommend refusing to treat Vikram when the relevant cost he would bear (his health) will not be borne by others. The financial costs, on the other hand, are such that if Vikram does not meet them, others must do so, and so the fairness–based justification could warrant increasing the payments Vikram must make over a non–responsible patient. Thus, responsibilisation could, and probably should, come in forms other than automatic treatment denial, such as taking responsibility as one factor among many in making prioritisation decisions, using it as a ‘tie–breaker’ (Segall, 2010, p. 69; Thornton, 2009), or increasing co–payments.

The second relevant response to the harshness objection notes that luck egalitarianism is an ideal theory. Segall (2013, pp. 178–179) suggests that responsibility–sensitivity only applies against a background of equal opportunity (see also Arneson, 1989, p. 86; Barry, 2006). Thus, where people face obstacles to equally responsible exercises of agency—either internal obstacles, such as lack of willpower, or external obstacles such as lack of time (Brink & Nelkin, 2013; Dworkin, 1981)—responsibilisation is unjustified. Non–egalitarian proponents of responsibility–sensitivity might take a sufficientarian version of this view, where what matters is that people have sufficient opportunity to choose well (Davies & Savulescu, 2020). As Knight (2015, p. 122) notes, though, this response seems less plausible against some paradigm cases of harshness, such as accidents caused by reckless driving.

I return to these responses shortly. It is worth reiterating, though, that the debate over harshness tends to focus only on the issue of the responsible individual taking on either what I called the ‘direct health costs’ of a choice, or the equivalent financial costs. There has been little prolonged discussion of the subsequent effects of a person having to take on these costs. This may artificially restrict the range of cases we should apparently worry about to those where we need only be concerned with either the health or immediate financial consequences of an agent's choices (though see Albertsen, 2015, p. 163).

So, the recursion problem is that holding people responsible for their health in ways that make them less likely to access care (the most obvious example being automatic treatment denial) makes more likely a negative cycle: the costs of being held responsible for an initial choice include worsened health, which in turn reduces the individual's ability to choose well by limiting either her internal resources or external resources. She is then more likely to make non–optimal choices and, *unless some principled reason is available against doing so*, to be held responsible again, potentially worsening her health further.

Proponents might object that my outline of the recursion problem ignores the first response considered above, that responsibilisation need not involve treatment denial. If the recursion problem only applies to the harshest interpretation of responsibilisation, it is not a significant problem. But while the recursion problem is starkest under extreme policies, it is not *only* automatic treatment–denial that raises it. Using responsibility as a ‘tie–breaker’ between patients competing for treatment, or as one feature among several which decides patients' places on a waiting list, will sometimes mean that patients face delays to treatment, with worsened or extended poor health and concomitant effects on opportunity. It may also be functionally equivalent to treatment denial if treatment must occur at a particular stage. Increasing financial charges could make treatment unaffordable for some or increase reluctance to seek treatment. Any form of responsibilisation that attaches conditions on access to healthcare has the potential to generate the recursion problem. My analysis does not apply, though, to pre–treatment forms of responsibilisation such as taxation.

Cavallero (2011) suggests that in actual societies, attempts to impose luck egalitarian policies in healthcare while leaving other social inequalities unchanged will likely worsen the latter, since those who are socially worse off are more likely to adopt *easily detectable* and *socially unaccepted* health–worsening habits. While some luck egalitarians (Roemer, 1998) advocate such piecemeal introduction of luck egalitarianism into policy, others explicitly reject it (Albertsen, 2015; Voigt, 2013) for this reason. But the recursion problem can occur even against a background of equal or sufficient opportunity, and even under more equitable behavioural targeting. The cyclical relationship between health and opportunity makes it plausible that, on average, health–promoting choices lead to further opportunities to make health–promoting choices, while health–undermining choices make it harder to choose well later. Indeed, this is an old worry about what Dworkin (1981, p. 209) calls ‘starting–gate’ views, where what matters is that people begin with equal (or otherwise fair) shares of resources, opportunity, or some other currency of justice (Chambers, 2009; Fleurbaey, 2002) and are then left alone. Thus, an initial decision to hold someone responsible in ways affecting their health may mean they end up very badly off, potentially even unable to exercise responsibility at all.

Stemplowska (2017) argues that luck egalitarians should consider whether a particular disadvantage resulted from a person's choice ‘only if acceptable payoffs attach to a given choice’. This means we should refuse compensation for disadvantage only if the costs of avoiding that disadvantage were greater than the costs of compensation. This provides a partial response to the recursion problem; cases where someone's worsening situation makes it increasingly costly for them to make healthy choices, such as the person who must choose between eating and heating, cannot be held substantively responsible.

However, where dwindling opportunities or poor health mean it becomes more difficult to make good choices, e.g., because it is harder to see what those choices are, or simply because one's motivation has been sapped, it may still be true that if one *were* to choose well it would not be costly to do so. Moreover, Stemplowska does not explicitly consider a key issue the recursion problem raises, where the increased cost of making healthy choices is *itself* a result of a prior choice under favourable conditions. Luck egalitarians must explain why, or at least when, such limitations on choice that are *foreseeably caused* by prior choice are not among the ‘chosen inequalities’ to which responsibility applies.

Some proponents of responsibilisation may bite the bullet. If people are responsible in their initial choice, and remain responsible in subsequent choices, they should bear the costs. This is clearly a coherent response. However, it is unclear why we should accept this role for responsibility. Particularly in cases where the costs to others of helping are significantly less than the costs to the patient if nobody helps, it really does seem excessively harsh to insist that I bear those latter costs to spare others the former. This is, of course, no more than a restatement of the harshness objection. But insofar as that objection resonates, those who support responsibilisation may seek a more moderate response to the recursion problem. That is, for those moved both by worries about harshness but also concerns about responsibility, it would be attractive to find a principled point at which to stop the cyclical decline in which some individuals may find themselves due to responsibilisation.

## 4 THREE SOLUTIONS

This section considers three possible solutions to the recursion problem, where a ‘solution’ is a proposal that would be attractive to someone who wants to retain some role for responsibility in healthcare, but finds the recursion problem a genuine worry.

The first such solution, suggested to me by Andreas Albertsen, depends on a particular interpretation of the core luck egalitarian commitment. I have presented luck egalitarianism as saying that whatever results from the properly situated exercise of choice is not thereby unjust. However, Albertsen suggests understanding the luck egalitarian commitment as saying that ‘our relative position should reflect our exercise of responsibility’. This would mean that at some point, a luck egalitarian could insist that an individual's position is no longer *proportionate* to their exercise of responsibility.

My view is that this is not what most luck egalitarians mean and is anyway not an attractive solution. Such a view implies not only that it is not unjust if some are worse off than others through their free choices, but that it *is* unjust if some are equally well off despite their free, reckless choices. For instance, if someone performs a risky action like smoking but does not suffer, a proportionality view seems to imply that this is unfair, since their well–being is not proportional to their exercise of responsibility. This is not a view that most luck egalitarians endorse (Lippert‐Rasmussen, 2016, pp. 75–76), though Albertsen has pointed out to me that some do endorse ‘symmetrical’ luck egalitarianism that condemns ‘arbitrary’ equalities (see Albertsen & Midtgaard, 2014 for references and defense).

I cannot see the attraction of such a view, at least without qualification (see also Segall, 2010, pp. 16–18). It seems to imply that if someone chooses badly (crossing the road without looking) but gets lucky and is unharmed, we should *actively ensure* they get the comparative disadvantage they ‘deserve’.^1^ My view is that such cases of lucky imprudence are generally cause for gladness. I accept, though, that there are possible variations on this theme. One might restrict proportionality to claims about when we should positively assist one another, rejecting proportionality if it would involve making people worse off when this benefits nobody. Indeed, this seems to be the basis of Albertsen & Midtgaard's view, since they support their symmetrical approach by saying that people may not call for compensation when they ‘could have easily avoided a disadvantage or inequality’. They thus oppose ‘equalizing measures’ in such cases, a weaker idea than the claim that relative positions should ‘reflect’ responsibility.

Still, there are three further problems. First, this view suffers from an indeterminacy that parallels objections to retributive punishment (Walen, 2020) in deciding what level of ill health is proportionate to a particular exercise of responsibility. It also presents a practical challenge to implementing it in a healthcare system: to know when to step in, we must know precise details about individual exercises of responsibility, which are unlikely to be available. This problem might be avoided by employing rules of thumb, e.g., that those who harm their health through addictive substances are less blameworthy than those who harm their health in other ways. But an overall estimation of the degree to which someone has exercised responsibility still requires a significant level of information.

Finally, while it provides *some* resistance to recursion, it is not clear whether this approach solves the problem. Albertsen & Midtgaard suggests that individuals may not demand ‘equalizing measures’ when these would make their position disproportionate to their exercise of responsibility. But it is possible for people to incrementally become very badly off through repeated bad choices, each of which is made more likely by the disadvantages resulting from their previous choices. Albertsen & Midtgaard appeal to the familiar idea of a ‘background of equal opportunities’; but their view does not seem to tackle the problem that this background quickly—and, by luck egalitarian standards, licitly—slips away.

A second approach to recursion is to borrow a popular solution to the harshness objection: an appeal to sufficiency. Roughly, ‘sufficientarians’ think distributive justice requires that people have ‘enough’. For instance, Shields (2016) emphasises the importance of the difference between being barely capable of autonomous decision–making and being incapable of it. Others focus on flourishing (Ram‐Tiktin, 2016), or contentment (Frankfurt, 1987). Some luck egalitarians suggest that as well as the demands of justice, there are distinct, sufficiency–*based* (but not ‘sufficientarian’) reasons to prevent people from becoming very badly off (e.g., Segall, 2010). As Nielsen (2013, p. 409) puts it, this involves ‘a separation of the realm of justice from that of basic needs’, which are covered by non–justice obligations, e.g., of beneficence.

Alternatively, one might adopt a pluralist approach to justice, with genuinely sufficientarian constraints on responsibility (e.g., Herlitz, 2019). Perhaps people should bear the burdens of their choices up to the sufficiency threshold, but others should shoulder burdens which would take them below that threshold. The plausibility of this approach depends on the threshold. As a view of justice, sufficientarianism has traditionally had two distinct goals. The first is to counter a worry about arbitrariness, finding a threshold where we should treat those just below it very differently than those just above it. This has led to identifying fundamental goods, such as the capacity for autonomy, as grounding the threshold, setting the bar low. But some sufficientarians want to demonstrate that among people who are above the sufficiency threshold, there are no further demands of justice. This often involves high thresholds, such as Crisp's (2003) comparison of the rich and ‘super–rich’, or Frankfurt's (1987) focus on contentment. Other sufficientarians adopt a pluralistic view, with separate thresholds serving each function.

This presents a challenge for a sufficiency solution to recursion. A low threshold, such as the bare capacity for autonomy, allows the recursion problem to progress significantly. Yet a high threshold reduces the scope of responsibility, perhaps beyond purpose. Consider, for instance, a policy which says that people should bear the costs of their own choices unless this made them discontented.

A plausible sufficientarian solution to the recursion problem thus needs to find a middling threshold which gives responsibility considerable sway, but which does not let people fall too far *and* which is not arbitrary. (Of course, the implausibility of a particular threshold in the context of responsibility does not entail its more general implausibility as a sufficientarian threshold). One option is competitiveness. Recall that one ingredient of the recursion problem is the fact that poor health makes individuals comparatively less competitive for opportunities, which in turn makes them more vulnerable to making choices that are bad for their health.^2^ A sufficiency–based response to the recursion problem might suggest that no individual should be made unable to compete against other similarly talented individuals. This would potentially allow for the idea that people should bear considerable costs from their choices but would not mean that people would become too badly off. Moreover, the threshold of competitiveness might be thought to have independent plausibility given the source of the recursion problem. A competitiveness threshold might be relative not to comparisons with everyone in a society, but only with those against whom one would ordinarily compete (Ben Shahar, 2018, p. 109).

This proposal also faces practical challenges. For instance, should we understand ‘those against whom one would ordinarily compete’ as referring to one's position in the actual world, in an ideally just society, or something else? (Chambers, 2009, pp. 379–380). Many people cannot develop their talents fully due to injustice, and so *could potentially* compete for more prestigious and rewarding positions than they do. If we take a person's actual competitiveness as our benchmark, this seems to ignore prior injustice. Yet *ideal* competitiveness seems extraordinarily difficult to estimate.

It is also unclear how to interpret ‘unable to compete’. In a highly competitive job market where applicants significantly outnumber positions, any change in circumstances might make you less competitive, and so it seems unrealistic to say that one must remain just as competitive before being held responsible as after. Yet it is not clear what a reasonable alternative might be: should we say, for instance, that after being held responsible an individual must remain ‘minimally’ competitive for the positions she could have reasonably expected to compete for before? If so, then we need to offer an account of what it means to be minimally competitive that can be operationalised in a healthcare system.

A third solution, which I also have space only to sketch, draws on the other potential justification for responsibilisation I raised at the beginning of the paper. While I have focused on the justification that others should not bear the costs of my choices, that second justification was that responsibilisation is justified by its making people more likely to behave responsibly in the future. The recursion problem applies to cases where instead of making better choices, people become more likely to make poor choices; though this is not necessarily because they have become ‘less responsible’. It may rather be because good choices become more difficult or costly.

McGeer (2019) uses the term ‘capacitation’ for the idea that holding people responsible will sometimes help them to be more responsible in the future. McGeer rejects desert–based views of responsibility. But it seems possible that someone who was motivated in part by the idea of a fair allocation of costs (a ‘distributive’ justification for responsibilisation) could also accept the idea of capacitation as an external constraint on the costs of responsibility, similar to the sufficiency–based constraints discussed above. Thus, even if *one* justification for responsibilisation is the distribution of costs, i.e., the idea that as far as possible people should bear the costs of their own choices, one might think our practices of holding people responsible should also help them *be* responsible.

On one interpretation of this requirement, capacitation provides a necessary condition that must be met to justify responsibilisation. Where responsibilisation will diminish a person's capacity for future responsibility, then even if otherwise justified on distributive grounds this counts strongly, perhaps even decisively, against holding this person substantively responsible (at least, in this way, or to this degree).

Another version of this approach sees any judgement of substantive responsibility as a prompt for two, parallel mechanisms. The first mechanism is distributive, where an individual is made to cover the relevant costs of their choice (this mechanism could itself be constrained by other considerations). The second mechanism is capacitating. Whereas the weaker version of the capacitation approach simply uses future responsibility as a limit on holding someone responsible, the stronger version involves the provision of *support* for the individual to make better choices in the future, even while insisting that they bear some costs of that decision.

This cannot apply in all cases; for instance, situations where one of two patients must die. Yet such cases are, arguably, not appropriate locations for responsibilisation anyway, *inter alia* because they place an unreasonable burden on healthcare workers (Davies & Savulescu, 2020, p. 425).

I lack the space to develop these possibilities in greater detail. My aim in this section has not been to defend a solution to the recursion problem—I am not convinced there necessarily is a satisfying one—but to identify and sketch potentially promising approaches to this problem from within a perspective which wants to hold on to responsibility without succumbing to the extended problem of harshness raised by the recursion problem.

## 5 CONCLUSION

The recursion problem is, in one sense, an expanded version of the harshness objection. However, it is not merely that. For I have argued that the recursion problem is such that many standard luck egalitarian responses (which I take to be roughly representative of the kinds of responses other proponents of responsibilisation might give) do not avoid the recursion problem with equal ease. ‘Harshness’ is not simply a one–off effect which influences only a person's health; it has the potential to become suffused throughout people's lives. Some proponents of responsibilisation may accept this, and I take nothing I have written to convince them that they should not. Others will already be unconvinced by responsibility, and so not regard this problem as an interesting addition to an already dead proposal. For the rest of us, though, the recursion problem raises a deeper challenge to the role responsibility might play in distributive justice.

## Footnotes

1 Some might think this conflates Albertsen's proposal with a *non–comparative* desert view. But the person who is hit by a car while unsafely crossing the road incurs disadvantages compared with others; an unconstrained version of the proportionality view need not insist that we ‘correct’ the resulting inequality by directly hurting the lucky individual, but does seem to imply we should ensure that they suffer the various disadvantages somehow.

2 My thanks to Tammy Harel Ben Shahar for this suggestion.
